# Broad Range Mid-IR Reflection Spectroscopy for Macroscale Standoff Hyperspectral Imaging of Paintings

**DOI:** 10.1021/acssensors.5c00865

**Published:** 2025-07-15

**Authors:** Francesca Rosi, Laura Cartechini, David Buti, Francesca Sabatini, Aldo Romani, Diego Sali, Xia Wu, Roland Harig, M. Cristina Tomassetti, Brunetto G. Brunetti, Costanza Miliani

**Affiliations:** † Institute of Chemical Sciences and Technologies ″Giulio Natta″−SCITEC, 9327 National Research Council-CNR, Via Elce di Sotto 8, 06123 Perugia, Italy; ‡ Institute of Heritage Science- ISPC, National Research Council-CNR, Area della Ricerca di Firenze Via Madonna del Piano 10, 50019 Sesto Fiorentino, Firenze, Italy; § Institute of Heritage Science- ISPC, National Research Council-CNR, Via Cardinale Guglielmo Sanfelice 8, 80134 Napoli, Italy; ∥ Centre of Excellence SMAArt c/o Department of Chemistry, Biology and Biotechnology, University of Perugia, via Elce di Sotto 8, 06123 Perugia, Italy; ⊥ Bruker Italia S.r.l. Unipersonale, Viale Vincenzo Lancetti 43, Milan 20158, Italy; # Bruker Optics GmbH & Co. KG, Rudolf-Plank-Str. 27, 76275 Ettlingen, Germany; ∇ Parco Archeologico di Cerveteri e Tarquinia, Piazza Cavour 1, 01016 Tarquinia, Italy

**Keywords:** degradation, paint binder, noninvasiveness, mid-IR spectroscopy, chemical imaging

## Abstract

The identification of the chemical composition of painting materials and their macroscale distribution is crucial for effective conservation, knowledge, and enhancement of our irreplaceable cultural patrimony. The noninvasive comprehensive characterization of both organic and inorganic components in paintings is currently achieved by mid-infrared (mid-IR) spectroscopy at single points or by chemical mapping via point-by-point scans over areas of square centimeters. However, the lengthy acquisition time required for mapping measurements makes it difficult to record chemical distribution images within a reasonable time frame. This limitation underscores the need for a hyperspectral imaging approach. To address this challenge, an advanced hyperspectral imager operating in the mid-IR spectral range (4000–800 cm^–1^) is presented here, providing compositional images of great value for the study and conservation of paintings. The system allows heritage scientists to record, within a few minutes, accurate distributions of inorganic and organic components, most importantly of degradation products, and it enables real-time monitoring of restoration intervention. The imager was first validated through mock-ups of known composition by direct comparison of spectra extracted from the hypercube data set with those acquired in the same areas by a portable single-point IR spectrometer. Then, insights into the nature and localization of original and degradation materials were provided for a precious Renaissance painting under restoration through in situ measurements. The results achieved put in evidence how the advances of mid-IR hyperspectral imaging represent a significant step ahead in the scientific examination of tangible cultural heritage.

## Graphical abstract

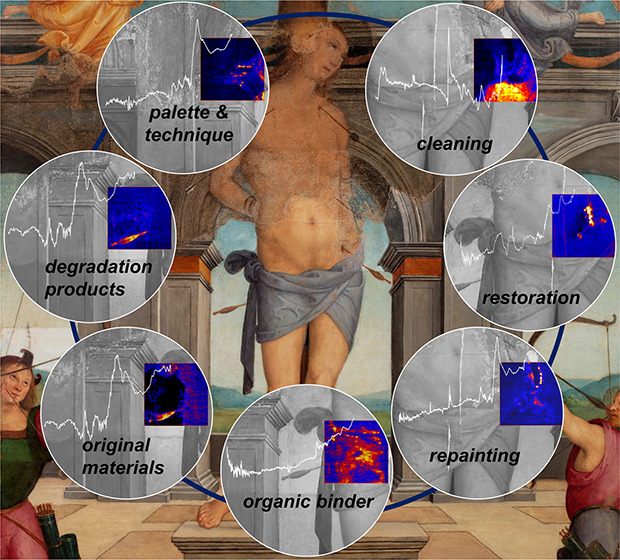

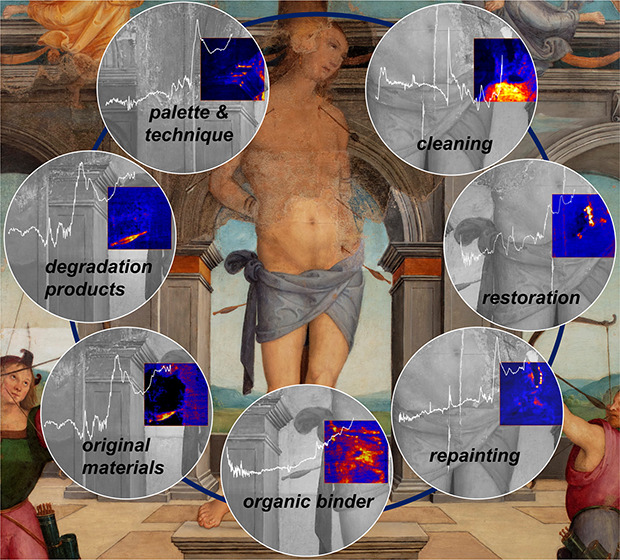

Heritage science research has seen impressive growth in the last years thanks to advancements in technologies and methods aimed at understanding tangible heritage by noninvasive spectroscopic analytical approaches. To answer the complex and challenging questions posed by art-historians, archeologists, curators, restorers, and conservation scientists, spanning across multidisciplinary fields, it is crucial to go beyond the knowledge of the chemical composition of materials and provide their distributions at the macroscale. Driven by these needs, in recent years, advancements in heritage science have been oriented toward the development and application of hyperspectral imaging/scanning systems.

Chemical imaging at the macroscale in heritage science can currently count on X-ray fluorescence scanning devices (MAXRF), showing the visible and invisible materials as elemental distribution maps.^1–3^ This information is generally integrated with molecular imaging spectroscopy from the visible (VIS) to the short-wave infrared (SWIR) range (400–2500 nm), enabling the identification of several pigments and the characterization of some organic binders through the observation of their electronic and vibrational (combination and overtone) bands.^4–6^ More recently, the introduction of macro X-ray diffraction (MAXRD) mapping^7,8^ expanded the obtainable information, providing the identification and localization of original and degradation materials, although with limitations in terms of long acquisition time and compositional information restricted to crystalline phases.

In this scenario, the highly diagnostic mid-infrared spectral range (4000–400 cm^–1^) has not been fully explored in portable and standoff hyperspectral imaging, although it is recognized that its inspection enables the identification of several organic and inorganic materials, including pigments, binders, and surface coatings (both traditional and synthetic polymers), and most of the common alteration and degradation products (both crystalline and amorphous phases).^9–13^ These potentialities led to successful applications also in support of restoration, particularly in the monitoring of cleaning.^9,12,14,15^

For all of these reasons, considerable efforts have been spent in recent years to develop and apply mid-IR hyperspectral imaging systems, although, to date, these applications are restricted only to a few studies. The first mid-IR hyperspectral imaging of a painting dates to 2013.^16^ This work unveiled several aspects of the chemical nature and the macroscale spatial distribution of constituent materials of a painting by Alberto Burri, despite the narrow operational spectral range (1440–900 cm^–1^). Similarly, following studies successfully exploited different cameras and acquisition modalities; however, again, they explored a limited mid-IR wavenumber range.^17–20^

In external reflection IR spectroscopy of a polychrome surface, spectral interpretation is challenging due to well-known spectral distortions arising from the collection of volume and surface reflections in a variable and unpredictable ratio.^9^ Access to a broader IR range enhances spectral identification, enabling cross-verifications through multiple bands that are not all visible or accessible within the previously mentioned narrow range (Table 1).

**Table 1:** Comparison of Mid-IR Imaging and Scanning Systems Applied in Heritage Science with the Broad Range Mid-IR Imager Presented in This Study

parameters	narrow range mid-IR imagers^16–20^	single-point scanning mid-IR system^21,22^	broad range mid-IR imager (this work)
spectral range	ca. 1300–800 cm^–1^	7000–400 cm^–1^	4000–800 cm^–1^
	ca. 3700–1800 cm^–1^		
total acquisition time for a 10 × 10 cm^2^ area	1–2 min	ca. 14h/28h	8 min
		(5/10 s per spectrum, step size 1 mm)	
spectral resolution	4 cm^–1^	4 cm^–1^	4 cm^–1^
lateral resolution	>0.5 mm	ca. 5 mm	0.7–0.8 mm at 1 m distance

To cover the full mid-IR range and profitably exploit the relevant analytical potentials of external reflection infrared spectroscopy, portable single-point IR spectrometers have been mounted on scanning systems for chemical mapping of small areas.^21,22^ Nevertheless, such measurements require long acquisition times (5–10 s per spectrum or point), which might render it difficult or impracticable to scan large artwork surfaces in museums or galleries (Table 1).

Herein, we present mid-IR hyperspectral chemical imaging (from 4000 to 800 cm^–1^) as obtained through an imager recently developed for noncontact in situ reflection measurements from IR optically thick surfaces. After preliminary validation tests on mock-ups, chemical images have been recorded on a painting by Pietro Vannucci, called *il Perugino* (1450ca.-1523). The results unveiled visible and invisible compositional details referring to original paints, retouching, restoration materials, and the state of conservation of the artwork.

## Experimental Section

### Mid-IR Hyperspectral Imager

A hyperspectral imager is a prototype developed by Bruker Optics in collaboration with the authors. It is an improvement of the commercial HI90,^23^ and it consists of a Michelson interferometer and a Stirling-cooled focal-plane-array (FPA) strained layer superlattice (SLS) detector, specially synthesized to allow for a satisfactory response in the mid-IR range. A plane-mirror interferometer is actively aligned, and the system is sealed and actively cooled. The maximum spectral resolution is 1 cm^–1^; however, all the measurements have been carried out at 4 cm^–1^, typically applied in IR external reflection mode in the 4000–800 cm^–1^ spectral range for heritage science applications. The full size of the FPA detector is 320 × 256 pixels; each pixel is 30 × 30 μm^2^. The field of view of an individual pixel is ca. 0.52 mrad, which corresponds to a lateral resolution of better than 1 mm (0.7–0.8 mm) at a 1 m working distance. A preview mode, operating like a thermographic camera, is generally applied to align and optimize the source before the hyperspectral acquisition with exploitation of the full size of the detector array. In this modality, no spectra are collected, i.e., no wavelength dispersion is present. For hyperspectral measurements, to allow the large number of signals to be better managed by the electronics, the system works in the so-called block mode. Namely, each block has a size of 176 × 22 pixels for a total of 8 blocks consecutively activated to cover the 176 × 176 pixels hyperspectral image for an area of ca. 11 × 11 cm^2^ at a 1 m working distance. The noise equivalent temperature difference (NETD) of the measurements with 16 coadds, 3 × 3 pixel averaging, in the spectral range from 1050 to 1150 cm^–1^, using an internal blackbody at a temperature of 300 K is 93mK. Reflectance spectra are calculated as the ratio between the mid-IR reflection data cube of the sample and that from a reference target (see Supporting Information-SI). To measure in reflection mode, the sample is illuminated by an infrared radiation source consisting of a globar (SiC) light source and a parabolic condenser mirror with a diameter of about 30 cm. The globar is installed on a motorized actuator that is used to adjust the working distance of the source from 1 m up to infinity at the defocused position. After each measurement, during data saving and fast Fourier transform (FFT), the actuator is automatically moved to defocus the source, or the source can be temporarily turned off to reduce the radiation exposure of the sample. The temperature and thus the radiation spectrum and intensity of the source can be tuned by changing the input electrical power. The intensity can also be attenuated by a manually adjustable aperture. The temperature of the source can be measured and monitored by a pyrometer installed in the center of the condenser. The maximum source temperature is 1000 °C. The acquisition time of a complete hyperspectral data cube is 8 min (with 70 μs of integration time and 16 coadds). In this time, ca. 7 min is necessary for acquiring the full FPA size scanning 8 blocks of 22 lines (22 × 176pixels), and the last minute is required for saving the data and FFT. The temperature increase at the painting surface was 6 °C, measured by a light meter (Elsec 775), with a source temperature of 800 °C (the set value for the investigation of the Renaissance painting). A schematic drawing of the measurement setup is visible in Figure S1A. The external light source is integrated on top of the interferometer (Figure S1A), and the entire system is installed on a track, dolly, and raiser system for easy handling and horizontal and vertical movement (Figure S1B).

### Data Processing and Image Reconstruction

The hyperspectral images are mostly processed using a linear filter with a Gauß kernel of 3 × 3 pixels with a width of 3 pixels (for each chemical image, the specific pixel processing is indicated in the corresponding caption). Reflectance difference images are created by plotting the difference between two reflectance values for every pixel. These two values are taken at the two wavenumbers of the boundaries of the diagnostic spectral region used to map a specific compound. The selected spectral ranges are reported in the figures’ captions for each presented image. Independent of the band shape (derivative-like, reflectance minimum, or *Reststrahlen*), the higher value is always set as the minuend and the lower as the subtrahend to obtain a positive distribution map representing the specific identified compound. Correlation maps are also created using the correlation algorithm available in the software of the camera, where endmembers, representative of the different materials, were considered only for the spectral range with the diagnostic spectral features. For each correlation map, the reference spectral range and the color bar, representing the level of correlation, are reported.

### Portable FT-IR Spectrometer

Point-analysis measurements were carried out with a portable Alpha-R spectrometer (Bruker Optics) equipped with a Globar Mid-IR source, a 30° interferometer, and a room-temperature DLaTGS detector. Sampling was carried out by an external reflectance module with specular optics (22°/22°); the sampled spot had a diameter of about 6 mm. A total of 128 interferograms were acquired in the spectral range 7500–375 cm^–1^ with a spectral resolution of 4 cm^–1^.

### Integrated Macro-XRF (MAXRF) and VIS–NIR–SWIR Scanner

MAXRF and VIS–NIR–SWIR measurements were performed with the scanner IRIS (Bruker). The system is equipped with a 10 W X-ray tube generator with the Rh anode. Measurements were performed with a tube voltage of 40 kV, a current of 100 μA, and a collimator of 1 mm. In the measurement head, an output and input optical fiber system is located for VIS–NIR–SWIR reflection measurements. The excitation source is a halogen lamp with fiber optic output and emission in the 380–2500 nm range. The detection system consists of two spectrometers to cover from 400 to 2500 nm with spectral resolutions of <2 nm (400–1000 nm) and <9.5 nm (1000–2500 nm). The X-ray beam can be collimated onto the sample surface at a diameter of 0.5, 1, or 2 mm, while the spatial resolution of VIS–NIR–SWIR measurements is ∼ 0.8 mm. The integration time is typically 30–50 ms per pixel.

### Laboratory Mock-Ups

The first laboratory mock-up consisted of a canvas painted in 2016 with different binder–pigment combinations. The canvas was grounded with a white titanium dioxide–calcium carbonate (TiO_2_–CaCO_3_) vinyl paint. Furthermore, ultramarine oil-paint mock-ups subjected to different artificial aging were analyzed to test the imager for the identification and imaging of degradation products.

## Results and Discussion

### Mid-IR Hyperspectral Imaging of Model Samples: Identification and Localization of the Paint Binder and Degradation Products

The imager was first validated through the examination of paint model samples with known composition to assess its ability to identify and map organic materials (paint binders, varnishes, and restoration materials) and degradation products. These research questions are of foremost importance and generally answered in a noninvasive way through single-point mid-IR measurements.

As a first validation test, hyperspectral imaging was carried out on a canvas mock-up (Figure 1A) made of pigments painted with two different binders (acrylic and vinylic synthetic resins). The results enabled the distributions of the two binders to be correctly mapped (Figure 1B,C). These maps were obtained as reflectance difference at the edges of the (almost) derivative-like bands characteristic of the acrylic [Figure 1D, 1159–1203 cm^–1^ ν­(C–O), yellow rectangle] and vinylic binder [Figure[Fig fig1]D, 1230–1280 cm^–1^, ν­(C–O–C), gray rectangle].^24^ To validate the quality of the spectra recorded by the hyperspectral camera, Figure 1D shows a good match between the two spectra extracted from the data cube on the squares painted with the two binders (# marks, in Figure 1), after averaging over 3 × 3 pixels, and the corresponding spectra acquired by the portable single-point IR system. For completeness and comparison purposes, single-pixel profiles (without any filtering and binning) are also reported in Figure 1D (red and black § marked lines). Background correction, for converting the hypercube reflection data into reflectance spectra of Figure 1D, was performed using a gray aluminum powder (Al powder) reference target (see the SI for a detailed description, Figures S2 and S3).

**Figure 1:**
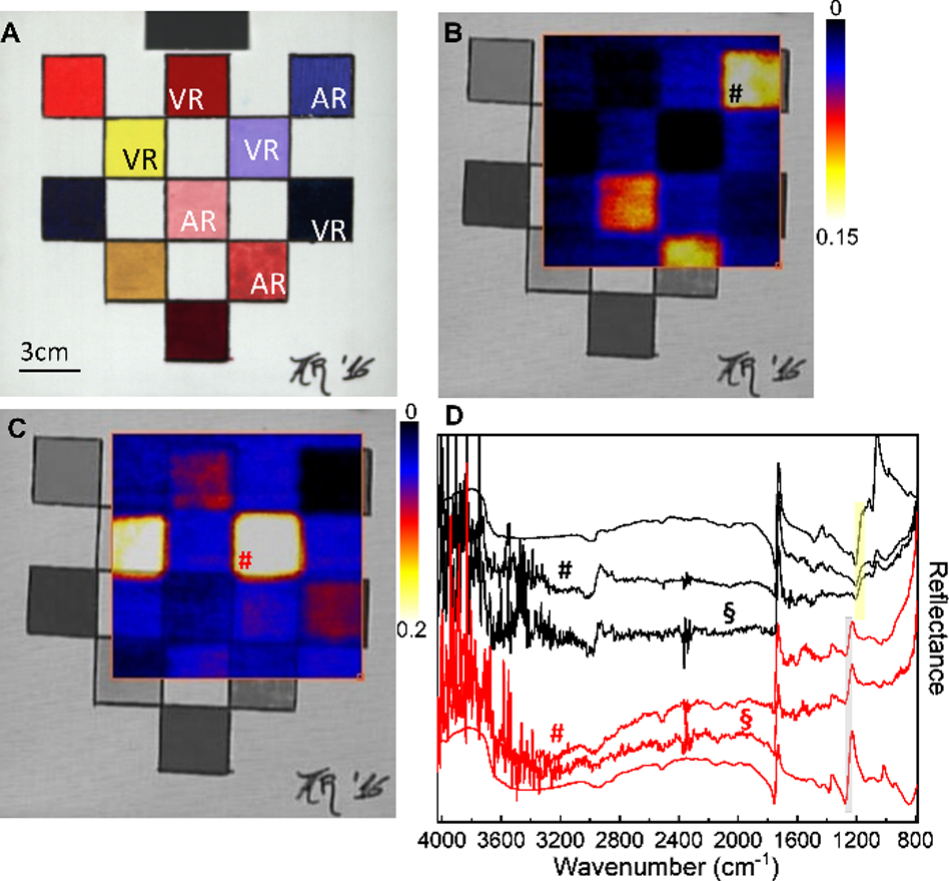
(A) Visible image of a canvas mock-up painted using different binders VR = vinyl resin and AR = acrylic resin; map of acrylic [(B), reflectance difference 1159 minus 1203 cm^–1^, ν­(C–O)] and vinyl binders [(C), reflectance difference 1230 minus 1280 cm^–1^, ν­(C–O–C)]; (D) reflectance spectra extracted from the hyperspectral data cube (3 × 3 pixel binning, # marks) in correspondence of acrylic and ultramarine blue (black line) and vinyl and Thénard’s blue (red line) paints compared with the spectra recorded with the portable single-point IR spectrometer (black and red lines without marks) on the same areas. Red and black § mark the single-pixel spectra extracted from the hypercube with no filtering and binning. Spectra are offset for the sake of clarity. Gray and yellow rectangles highlight the spectral ranges used for the reconstruction of the images shown in panels (B) and (C), respectively.

As a second validation test, degradation was assessed by performing the hyperspectral analysis on three identical ultramarine blue oil paint mock-ups that underwent different treatments (Figure 2).

**Figure 2:**
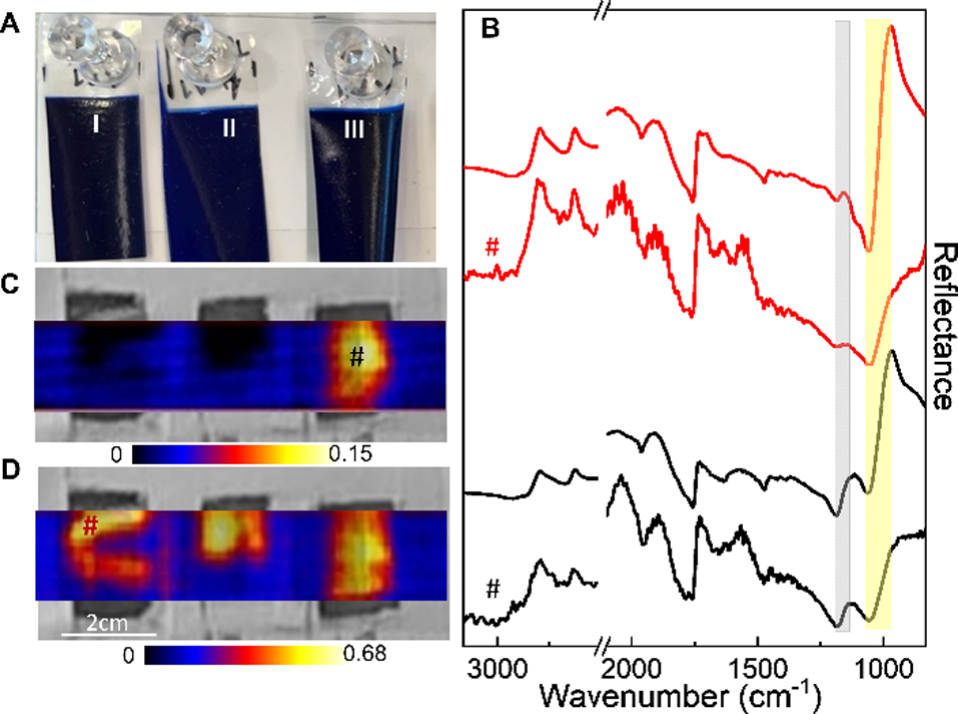
(A) Visible image of the mock-ups made of ultramarine blue oil paint applied on mylar foils (I unaged, II 100% RH and 65 °C aged, III SO_2_ aged); (B) reflectance spectra extracted from the hyperspectral data cube (3 × 3 pixel binning, # marks) in correspondence of the unaged (red line) and SO_2_ aged (black line) samples compared to the respective spectra recorded with the portable single-point IR spectrometer (black and red lines without marks). Map of sulfates [(C), reflectance difference 1134 minus 1180 cm^–1^, ν_3_(SO_4_^–2^)] and ultramarine blue [(D), reflectance difference 860 minus 1068 cm^–1^, ν_as_(Si–O)]. Gray and yellow rectangles highlight the considered spectral ranges used for the reconstruction of the images shown in panels (C) and (D), respectively.

The first one was left unaged (Figure 2A,I), the second exposed to 100% RH and 65 °C (Figure 2A,II), and the third incubated with SO_2_ vapor (Figure 2A,III). The spectra extracted from the data cube of the unaged (red line) and SO_2_ aged (black line) mock-ups are shown in Figure 2B (marked with #). The good agreement between these spectra and those recorded on the corresponding areas by the single-point IR spectrometer is also shown in Figure 2B (black and red lines without marks). The spectra from the SO_2_ aged mock-up clearly show the ν_3_ (SO_4_^–2^) *Reststrahlen* band^25^ of the newly formed sulfate (Figure 2B, black lines, gray rectangle 1134–1180 cm^–1^) due to degradation. The mid-IR mapping images further evidence that sulfate formation is restricted to the SO_2_-aged sample (Figure 2C). The spectral profiles of the 100% RH and 65 °C aged sample are reported in Figure S4 (imager versus single-point spectrometer, blue lines). The comparison with the corresponding unaged sample (red lines) underlines the formation of oxalates (weak band at 1315 cm^–1^)^10^ and the diminishing of the band assigned to ν­(C–O–C) at ca. 1100 cm^–1^ of the siccative oil, overcome by the strong ν­(Si–O) mode of the silicate-based pigment ultramarine.^25^ By mapping this latter signal (Figure 2B, yellow rectangle), the ultramarine blue pigment can be displayed (Figure 2D).

### Mid-IR Hyperspectral Imaging of the Renaissance Painting “Il Martirio di San Sebastiano” by Il Perugino

After the preliminary tests, measurements were carried out in situ on the Renaissance painting “*Il Martirio di San Sebastiano”*, 1518, by Pietro Vannucci, known as *Il Perugino*. The painting, conserved at the Galleria Nazionale dell’Umbria (Figure S5), was at that time under restoration. The goal of the hyperspectral measurements was to answer the three main questions posed by the restorers: (i) identification of the varnish and monitoring of the cleaning; (ii) identification of original materials; and (iii) assessment of the painting conservation state. Following the analytical protocol of such studies, a noninvasive multimodal approach was adopted, integrating the mid-IR imaging study with elemental MAXRF and molecular reflection-mode spectroscopy from VIS to mid-IR (Figures S6–S9).

Figure 3A depicts the 11 × 11 cm^2^ area (red square) at the interface between a cleaned and uncleaned portion of the painting, analyzed with the imager to answer question (i).

**Figure 3:**
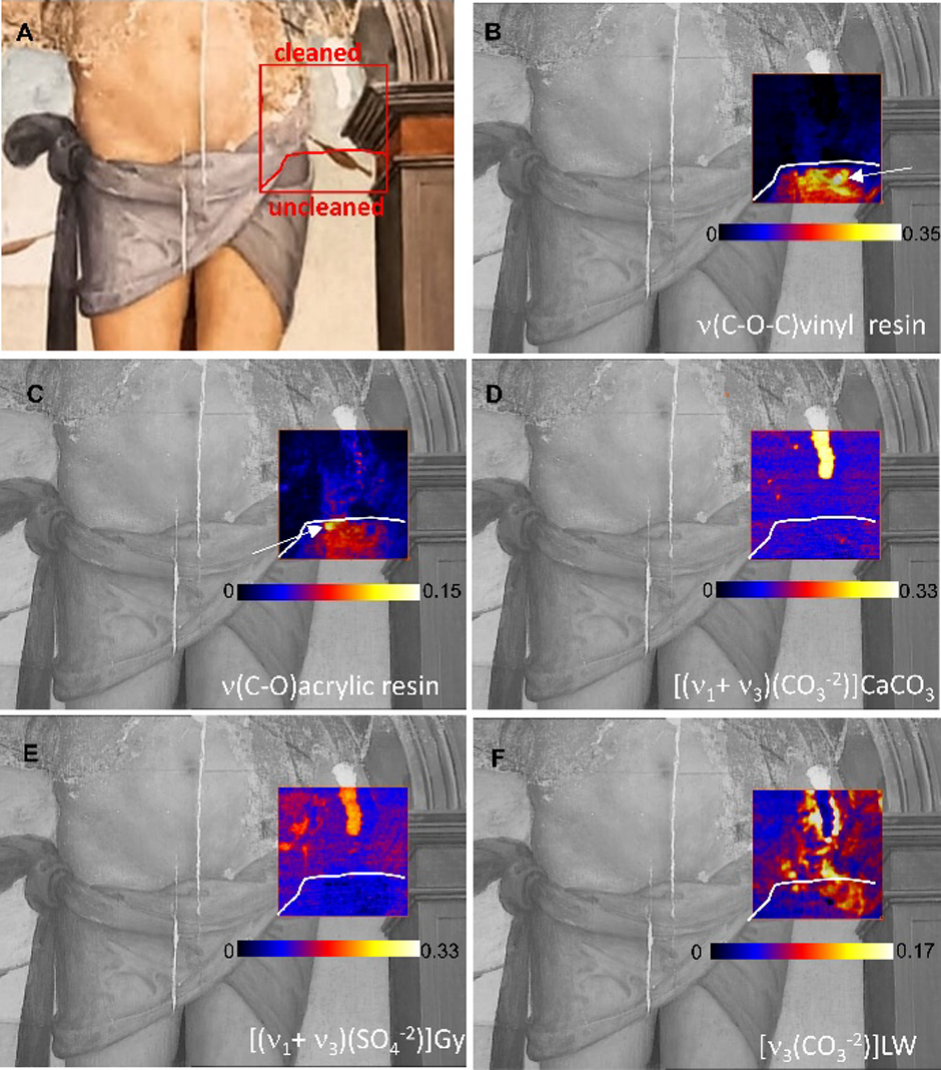
(A) Visible image of a portion of the panel painting “Martirio di San Sebastiano”, 1518, the red rectangle highlights the area investigated with the imager (ca. 11 × 11 cm^2^); map of (B) vinyl resin [reflectance difference 1230 minus 1280 cm^–1^, ν­(C–O–C)] and (C) acrylic resin [reflectance difference 1140 minus 1200 cm^–1^, ν­(C–O)]; (D) calcium carbonate reflectance difference 2517 minus 2440 cm^–1^, ν_1_+ν_3_(CO_3_^–2^)]; (E) gypsum, Gy [reflectance difference 2138 minus 2060 cm^–1^, ν_1_+ν_3_(SO_4_^–2^)]; (F) lead white, LW [reflectance difference 1387 minus 1500 cm^–1^, ν_3_(CO_3_^–2^)]. White arrows indicate the positions of the extracted spectra reported in Figure 4.

Figure 3B,C shows the distribution maps of acrylic and vinyl resin identified in the uncleaned portion of this area. The images were obtained from the reflectance difference at the edges of the two derivative-like bands at 1230–1280 cm^–1^ [ν­(C–O), vinyl resin] and at 1140–1200 cm^–1^ [ν­(C–O–C) cm^–1^, acrylic resin],^24^ as indicated in Figure 4 by yellow and green rectangles, respectively. The comparison of two spectra extracted from the hypercube (Figure 4, * marks) with reference spectra of vinylic and acrylic resins recorded by the single-point IR validates the results of the imager. The spatially resolved imaging capability allows for pointing out the distinct localization of the two varnishes in the uncleaned area, otherwise codetected by the single-point IR system (Figure 4, gray line), as well as some varnish residues in the cleaned areas after the intervention.

**Figure 4:**
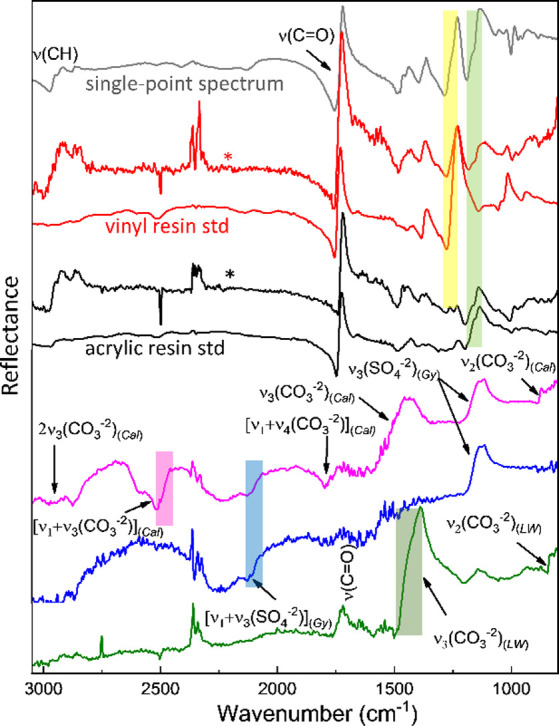
Spectra extracted from the hyperspectral data cube (3 × 3 pixel binning, * marks) with the specific spectral range used for mapping of Figure 3 highlighted by rectangles: vinyl resin (red line, yellow rectangle), acrylic resin (black line, green rectangle), gypsum (blue line and rectangle), calcium carbonate (magenta line and rectangle), and lead white (dark green line and rectangle). Single-point external reflectance IR spectra acquired on vinylic and acrylic reference samples (red and black lines, respectively) and in correspondence with the uncleaned area of the painting (gray line) are also reported. Spectra are offset for clarity. The main vibrational modes of the different materials are also indicated. Cal = calcium carbonate, LW = lead white, and Gy = gypsum.

The variable content of the two resins over the examined surface, underlined by the images of Figure 3B,C, is associated with coating materials applied during the recent restorations dated ca. 1960 and 1994–95 (vinyl and acrylic resin, respectively; see also the SI).

Notably, this finding allowed us to chemically and spatially differentiate between the two varnishes, which have different solubility properties, and thus to more accurately monitor the ongoing cleaning intervention.

In this area, further information concerning the conservation history of the painting was obtained. Indeed, maps of Figure 3D,E show that calcium carbonate and gypsum were used as fillers in the lacuna during the intervention dated 1994–95. The map of Figure 3E shows that gypsum is also revealed in areas where the ground layer of the original painting emerges due to the abraded surface. Finally, the distribution map of lead white, reported in Figure 3F, shows that this pigment, from either the original paint layer or the earliest repainting (see the SI), was extensively used to lighten the tones. During the ongoing cleaning, the retouched pigment was found to have accumulated in the cavities at the edges of the lacuna. Figure 4 reports the representative spectra extracted from the mid-IR hypercubes. For each spectrum, the vibrational modes considered for the mapping of Figure 3 are highlighted. The spectra from the abraded areas and lacuna clearly exhibit the intense combination bands of gypsum (both blue and magenta lines) and calcium carbonate (magenta line) that were fruitfully exploited for the identification and localization of these materials.^9,25^

Further chemical imaging results, obtained on another area, corresponding to a cleaned detail of the painting, are reported in Figure 5. Here, chemical images put in evidence the presence and localization of (a) some original materials (answering to question (ii)) and (b) degradation to calcium oxalate (addressing point (iii)).

**Figure 5:**
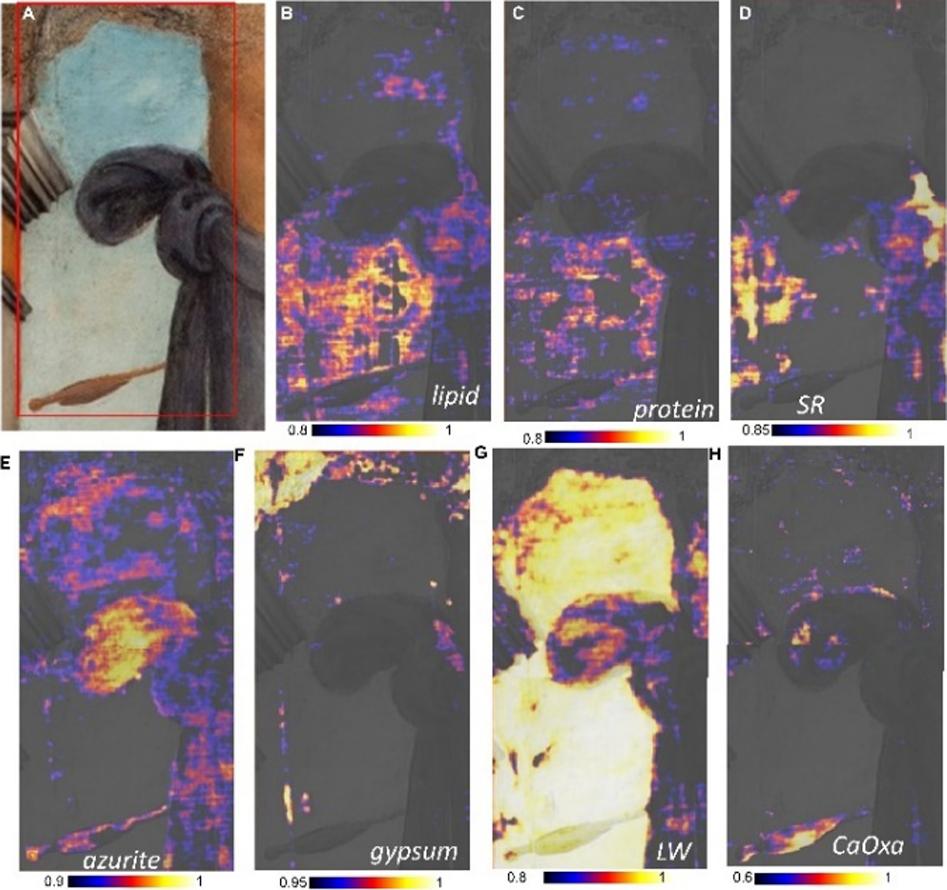
(A) Visible image of a portion of the panel painting “*Martirio di San Sebastiano*”, 1518, the red square highlights the area investigated with the camera (ca. 11 × 22 cm^2^); correlation images representing the distribution of (B) lipidic component [1650–1770 cm^–1^ ν­(C=O)]; (C) protein (1495–1665 cm^–1^ amide I and II bands); (D) synthetic varnish (SR) [1690–1790 cm^–1^, ν­(C=O)]; (E) azurite (Az) [1440–1660 cm^–1^ ν_3_(CO_3_^–2^)]; (F) gypsum (Gy) [1050–1200 cm^–1^ ν_3_(SO_4_^–2^)]; (G) lead white (LW) [1367–1490 cm^–1^ ν_3_(CO_3_^–2^)]; and (H) calcium oxalate (CaOxa) [1300–1360 cm^–1^ ν_s_(CO)]. Color bars represent the correlation values with respect to the reference profiles for the different compounds (reported in Figure 6). Images were processed by a linear filter with a Gauß kernel of 4 × 4 pixels.

In Figure 5A, the visible image of the examined area, corresponding to two adjacent squares of 11 × 11 cm^2^ each, is reported. Figure 5B–H shows the corresponding compositional maps obtained by applying a correlation algorithm to the recorded mid-IR hyperspectral cubes. The identified and mapped compounds are lipidic and proteinaceous components, synthetic varnish, azurite, lead white, gypsum, and calcium oxalate. The ability of the presented system to access a wide portion of the mid-IR range was critical for achieving these results, especially compared to previous narrow-band mid-IR imagers (Table 1).

The spectra of the data cubes exploited for the correlation mapping are reported in Figure 6.

**Figure 6:**
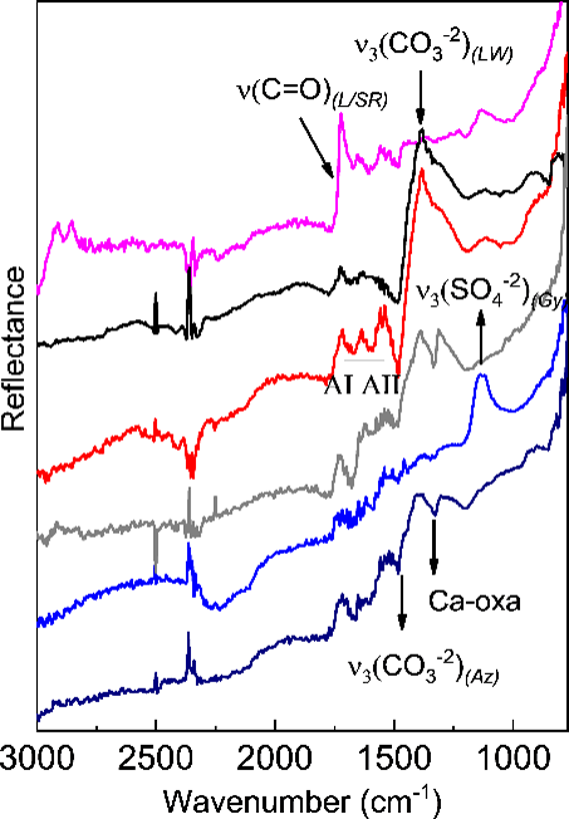
Reference profiles extracted from the mid-IR hypercube (4 × 4 pixel binning) with the vibrational modes considered for the correlation images indicated: synthetic resin (magenta line); lead white and lipids (black line); proteins (red line), calcium oxalates (gray line); gypsum (blue line); and azurite (dark blue line). Spectra are offset for clarity. LW = lead white, Gy = gypsum, L = lipid, SR = synthetic resin, AI = amide I, AII = amide II, Az = azurite, Ca-oxa = calcium oxalate.

The first relevant result is that lipid and proteinaceous components show an evident colocalization (Figure 5B,C). This suggests a whole egg *tempera* painting technique or *tempera grassa* painting technique (oil in a mixture with proteins). The proteinaceous component was mapped (C) by correlation analysis using the diagnostic spectral range of 1495–1665 cm^–1^ corresponding to amide I and II bands (Figure 6, red line). The lipidic component’s distribution in B was instead mapped by exploiting the carbonyl stretching from 1650 to 1770 cm^–1^ (Figure 6, black line). In the painting area under investigation, synthetic varnish residues (left over from incomplete cleaning, as discussed above) also contribute to the carbonyl stretching band (Figure 6, magenta line). However, these two contributions have been distinguished thanks to the different spectral profiles obtained in the reflection modality, namely, (a) a positive peak in the 1650–1770 cm^–1^ range for the lipid component mainly responding with volume reflection and (b) a derivative-like spectral shape in the region 1690–1790 cm^–1^ for the overlying varnish, responding as an optically flat surface.^9^

The differentiation of the varnish from the lipidic component found significant support by single-point IR measurements, which confirmed the presence of the lipid component via the typical doublet at 4300 cm^–1^ assigned to the (ν+δ)­(CH) mode of lipids^24^ (see Figure S7B).

The occurrence of *whole egg tempera* or *tempera grassa* (at least in the examined areas) may be considered surprising for a painting dated 1518, a period in which Perugino exclusively used oil as a binder. However, historical documentation^26^ suggests that this painting was begun in previous years and remained in the workshop for a long period before being completed and delivered to the client in 1518. This indication is further supported by the observation that in this painting, all of the brown areas are characterized by a brown earth pigment, which does not contain zinc impurities. On the contrary, Perugino, in his late period, after his return to Perugia around 1510 (following the closure of his workshop in Florence), used to paint with a brown earth pigment rich in zinc up to 20–25% wt.^27^

The map of Figure 5F evidences the presence of gypsum in the widely abraded areas of the sky and upper portion of the architecture, as well as in other scattered abrasions and scratches. Lead white (Figure 5G) is present in the lighter tonalities of the sky and, to a lesser extent, in the brighter shades of the loincloth and Saint’s body.

Access to the antisymmetric stretching region of the carbonate-based pigments^25^ made possible to reveal the use of azurite in the sky, in the blue–violet martyr’s loincloth and, surprisingly, in correspondence with the arrow detail (Figure 5E), although the Cu MAXRF map did not show evident signals of copper in that area (Figure S6I). VIS–NIR reflection spectroscopy corroborated this finding, indicating azurite (Figure S8A, purple line) as a shadow on top of the light brown paint. The azurite layer is too thin to be contrasted in the MAXRF Cu-map; however, it is, instead, easily detectable in the VIS and mid-IR spectral windows, with these ranges being more sensitive to the most superficial layers.

Further information on the painting materials used by Perugino is reported in Figure S6 and in the Supporting Information. Here, the MAXRF elemental maps and the mid-IR image show results in substantial agreement with those of Figure 5, confirming the presence of (a) gypsum in lacunas, abrasions, and scratches (see Ca–K MAXRF distribution), (b) lead white of the *imprimitura* and mixed with other pigments to create light tones (see Pb-L map), c) ochre-based pigment rich in kaolin in the brown tonalities (see Fe–K, Si–K, MAXRF, and mid-IR kaolin maps), (d) cinnabar, in the flesh tones (see Hg-L), and finally, (e) azurite (see Cu–K), whose presence is found in the sky and loincloth. The absence of copper on the arrow has already been commented.

Notably, the singular presence of Mn all along the loincloth area suggests the use of a red lake mixed with azurite to produce a bluish-violet hue visible to the naked eye. Indeed, it is known that red lakes were traditionally applied by Perugino mixed with transparent glass powder (containing manganese).^28^ In the present case, the absence of red pigment signals to produce the bluish-violet hue with azurite (Hg and Fe are absent in the loincloth; see Figure S6) points toward the application of a red lake mixed with azurite. On the contrary, no significant indications are present to support that a red lake was laid on the arrow tail or in the architecture, where Mn is revealed. Here, the colocalization of Fe and Mn suggests a more probable use of umber, a dark brown earth containing both elements.

Another important result achieved in this study is the identification and localization of calcium oxalate (Figure 5H). The identification of this degradation has been carried out through its typical ν_s_(CO) band at about 1320 cm^–1^, visible in Figure 6 gray line.^29^ The localization has been drawn by considering the correlation spectral range between 1300 and 1360 cm^–1^. The resulting map (Figure 5H) underlines the localized presence of calcium oxalate in correspondence with the arrow tail composed of a brown iron-based pigment with Mn, rich in Ca and containing kaolin, with an additional thin layer of azurite (MAXRF, VIS–NIR and mid-IR imaging in Figures 5, S6, S8, and S9). Interestingly, Ca oxalate is also present, though to a lesser extent, in the thick bluish-violet shadowed paints of the martyr’s loincloth (Figures 5H and 6, dark blue line, and Figure S9). The localized distribution of Ca oxalate is also supported by the elemental distribution maps of Ca–K (Figure S6), evidencing a satisfactory spatial correlation between the elemental and molecular chemical imaging results.

Generally, metal oxalates are degradation materials relatively common in works of art, although their origin remains unclear. In indoor environments, the more probable cause of oxalate formation in painting is the degradation of organic substances contained in the paint film or overimposed on it. The degradation produces oxalate ions, which then combine with calcium from the paint materials or surface deposits. Given that oxalate formation in paintings shows a large variability in the occurrence, it is likely that different mechanisms can be operative according to different material contexts and the conservation history of the painting.

## Conclusions

The new instrumentation here presented records reliable mid-IR hyperspectral data in a wider wavenumber range (4000–800 cm^–1^) than in previously available hyperspectral systems and with acquisition times sensibly lower than those required by the current scanning methods (time gain factor: 40).^21,22^

The device extends the obtainable hyperspectral analytical information, enforcing the toolbox of analytical methodologies available to researchers for frequent and expeditive applications of preventive conservation in museums, for the in situ monitoring of restoration interventions, and, more generally, for heritage science studies.

For example, an interesting finding of this study has been the definite and specific localization of calcium oxalate degradation. This is not the first case where an enhanced propensity of oxalate formation in areas of specific composition is found.^10,29,30^ However, in all cases, no definite conclusions about the causes of formation were achieved. In the near future, thanks to the fast analytical response of mid-IR hyperspectral imaging, information on oxalate distributions in paintings is expected to become increasingly available, opening new horizons in better understanding the causes and mechanisms of this degradation.

A notable diagnostic limitation of mid-IR spectroscopy, stemming from its high surface sensitivity, is the presence of a varnish that can hamper the mid-IR radiation from probing the underneath composition. However, during the initial stages of restoration, varnish layers are typically removed, effectively overcoming this limitation, as it occurred in the present study.

A particular advantage, offered by the hyperspectral imager of this work, lies just in the field of restoration for monitoring and optimizing interventions. In fact, prior to restoration, a thorough understanding of the nature and distribution of materials is crucial to support decision-making and the selection of the most appropriate methods of intervention, ensuring precise and effective operations. This is generally achieved by external reflection IR spectroscopy. Within this context, the substitution of single-point spectra with promptly available mid-IR images, which visualize the distribution of unwanted materials or their residues during cleaning, further enhances the precision, selectivity, and efficiency of the work.

Improvements of the hyperspectral mid-IR imager described here are currently ongoing. Efforts are directed to optimize the illumination setup for improving the spectral quality at a high wavenumber while minimizing the heating of the investigated surface. To further decrease the acquisition time, experiments at a lower spectral resolution (now 4 cm^–1^) will be performed.

Improvements are also ongoing in the treatment of the hyperspectral data cubes, here simply elaborated through univariate analysis. It is expected that a deeper examination of the hyperspectral data through state-of-the-art data elaboration tools and automated material classification or machine-learning-assisted spectral analysis will allow for extracting more extended information, enhancing analytical knowledge and the visualization of the critical compositional information.

## Supplementary Material



## Notes

Mid-IR imager, optimization of the background correction and imaging of model samples (Figures S1–S4); Renaissance painting “*Il Martirio di San Sebastiano*”, art-history, conservation history, and complementary analytical information from the multitechnique noninvasive approach (Figures S5–S9) (PDF)

## ○

Department of Earth and Environmental Sciences, University of Milano-Bicocca, Piazza della Scienza 1, 20126 Milan, Italy

## Abbreviations

IR: infrared
MAXRF: macro X-ray fluorescence
VIS: visible
NIR: near-infrared
SWIR: short-wave infrared
MAXRD: macro X-ray diffraction
FPA: focal plane array
SLS: strained layer superlattice
NEDT: noise equivalent temperature difference
FFT: fast Fourier transform
VR: vinyl resin
AR: acrylic resin
Gy: gypsum
LW: lead white
Cal: calcium carbonate
SR: synthetic resin
Az: azurite
Ca-Oxa: calcium oxalate
L: lipid
AI: amide I
AII: amide II
