# Supplementary material for: Broad Range Mid-IR Reflection Spectroscopy for Macroscale Standoff Hyperspectral Imaging of Paintings
Source: ACS Sens. 2025 Jul 15;10(10):7334–42. doi: 10.1021/acssensors.5c00865 (PMC12560125; doi:10.1021/acssensors.5c00865)
Supplement: Supplementary file 1 [file se5c00865_si_001.pdf]

Supporting Information

Broad range mid-IR reflection spectroscopy for macro-scale standoff hyperspectral imaging of paintings

Francesca Rosi[§*], Laura Cartechini[§*], David Buti[‡], Francesca Sabatini[§†], Aldo Romani[#§], Diego Sali[‖], Xia Wu[⊥], Roland Harig[⊥], Maria C. Tomassetti[∇], Brunetto G. Brunetti[§#], Costanza Miliani[‡]

*francesca.rosi@cnr.it laura.cartechini@cnr.it

[§] Institute of Chemical Sciences and Technologies "Giulio Natta" – SCITEC National Research Council-CNR via Elce di Sotto 8, 06123 Perugia, Italy

[‡] Institute of Heritage Science- ISPC, National Research Council- CNR, Address 1. Area della Ricerca di Firenze Via Madonna del Piano 10, 50019 Sesto Fiorentino, Firenze, Italy; Adress 2. Via Cardinale Guglielmo Sanfelice 8, 80134 Napoli, Italy

[#] Centre of Excellence SMAArt c/o Department of Chemistry, Biology, and Biotechnology, University of Perugia, via Elce di Sotto 8, 06123 Perugia

[‖] Bruker Italia S.r.l. Unipersonale, Viale Vincenzo Lancetti 43. Milano 20158, Italy

[⊥] Bruker Optics GmbH & Co. KG Rudolf-Plank-Str. 27, 76275 Ettlingen, Germany

[∇] Parco Archeologico di Cerveteri e Tarquinia Piazza Cavour 1, 01016 Tarquinia, Italy

Reflection to reflectance conversion: background correction

Previous applications of imagers working in the mid-IR region were restricted to ca. 1300 cm^{-1} as upper wavenumber limit. In those systems, radiometric calibration was applied to obtain the brightness temperature and/or the emission profiles used for material identification and chemical map reconstruction [1-2]. For the present imager, designed to explore the broad mid-IR range (moving upward the limit of 1300 cm^{-1}), it has been necessary to find the most suitable background reference target to compensate for the optical response of the spectrometer and the environmental water vapour absorptions as done in single-point external reflection mid-IR devices.

The background correction was optimized by testing reference materials with different reflectance to represent the variability of the optical properties of the investigated cultural heritage surfaces. In the case of paintings, the best results were obtained on a grey diffuse reference plate (made of aluminium powder and black coating applied on the surface with an organic undercoat, dimensions ca. 20x20 cm^2) and an Infragold target (ca. 20x20 cm^2, a gold mirror with a rough surface which reflects light diffusively). Figure S2 shows the reflectance spectra of the two targets, acquired by the single-point IR spectrometer against a flat gold mirror. Both the spectra show an almost flat behaviour along the IR range. The signals of the organic undercoat of the Al-powder reference plate show weak reflectance (Figure S2, red line) that do not substantially affect the background correction and the chemical imaging results (as will be demonstrated below).

The reflectance spectrum of the Infragold target (Figure S2, black line) is mainly constant with a slight greater drift below 1000 cm^{-1} possibly due to a stronger scattering of the diffusive surface at longer wavelengths.

We corrected the hyperspectral cube recorded on the canvas paint mock-up (Figure S3) using either the Al-powder or the Infragold references to evaluate the conversion from reflection to reflectance. The overall comparisons of the obtained maps for the acrylic and vinylic binders (Figures S3 B-E) shows similar results. Nevertheless, slight differences are observed in the corresponding spectral profiles (Fig. S3 F). More in detail, the comparison of spectra extracted from the squares painted with ultramarine blue and the acrylic resin (Primal-acrylic resin, 1) and Thénard's blue and the vinylic resin (Vinavil-vinylic resin, 2) shows how the use of the Infragold target generates profiles with a lower signal to noise ratio in correspondence of the water vapour absorption (1600-1300 cm^{-1}) and a larger drift in the lower wavenumber range with respect to the Al-powder grey target. For these reasons, the Al-powder grey target was finally selected for the reflectance conversion of the hypercubes presented in this paper.

The Renaissance painting "Il Martirio di San Sebastiano" (1518, Il Perugino)

A detail of the Renaissance painting "Il Martirio di San Sebastiano" (1518, Il Perugino) is reported in Figure S5. The painting, conserved at the National Gallery of Umbria, was under restoration during the present analytical campaign.

According to the restorers, the painting was subjected at least to four previous interventions, two of which are documented and dated to 1963 and 1994-95 and included mainly cleaning, retouching, removal of previous re-paintings, and varnishing.

The older two (it is not excluded that the first one was carried out already a few years after the painting completion) were characterized by widespread re-paintings [3]. The current intervention was aimed at cleaning the oxidized varnish and removing the most recent re-paints, to harmonize the original paint layer with the ancient makeover and thus making it possible to exhibit the Perugino panel painting again.

Modern white pigments (ZnO, $BaSO_4$ and Ti-based) define the composition of the second undocumented intervention. Pigments as azurite and lead white were detected in both original Perugino's paint and the oldest intervention. Figure S6 shows the elemental maps obtained by MAXRF scanning a portion of the painting (largely original) which underline the presence of: lead (B, lead white used for the "*imprimitura*" and for the lighter shades mixed with other pigments); calcium (C, as calcium carbonate and gypsum in lacunas and abraded areas of the painting); iron, potassium and silicon (D, E and G belonging to an ochre pigment rich in kaolin, as underlined by the mid-IR map, F) and manganese (H, due to umber or uncolored glass powder, usually added by Perugino to lakes to accelerate the oil drying [4,5]; copper (I, as azurite, present in the blue-violet paint of the loincloth most probably mixed with a red lake, and in the sky); and mercury (cinnabar, J).

References

[1] Rosi, F.; Miliani, C.; Braun, R.; Harig, R.; Sali, D.; Brunetti, B. G.; Sgamellotti, A. Noninvasive analysis of paintings by mid-infrared hyperspectral imaging. Angew. Chem. Int. Ed. 2013,20, 5258-5261.

[2] Gabrieli, F.; Dooley, K. A.; Zeibel, J. G.; Howe, J. D.; Delaney, J. K. Standoff Mid-Infrared Emissive Imaging Spectroscopy for Identification and Mapping of Materials in Polychrome Objects. Angew. Chem. Int. Ed. 2018,57, 7341-7345.

[3] https://catalogo.beniculturali.it/detail/HistoricOrArtisticProperty/1000016306

[4] Spring M. In *The Painting Technique of Pietro Vannucci, Called il Perugino*, Quaderni di Kermes, Nardini Ed.: Florence, 2004; pp 17–24.

[5] Seccaroni, C.; Moioli, P.; Borgia, I.; Brunetti, B.G.; Sgamellotti, A. In *The Painting Technique of Pietro Vannucci, Called il Perugino*, Quaderni di Kermes, Nardini Ed; Florence, 2004; pp 29-41.

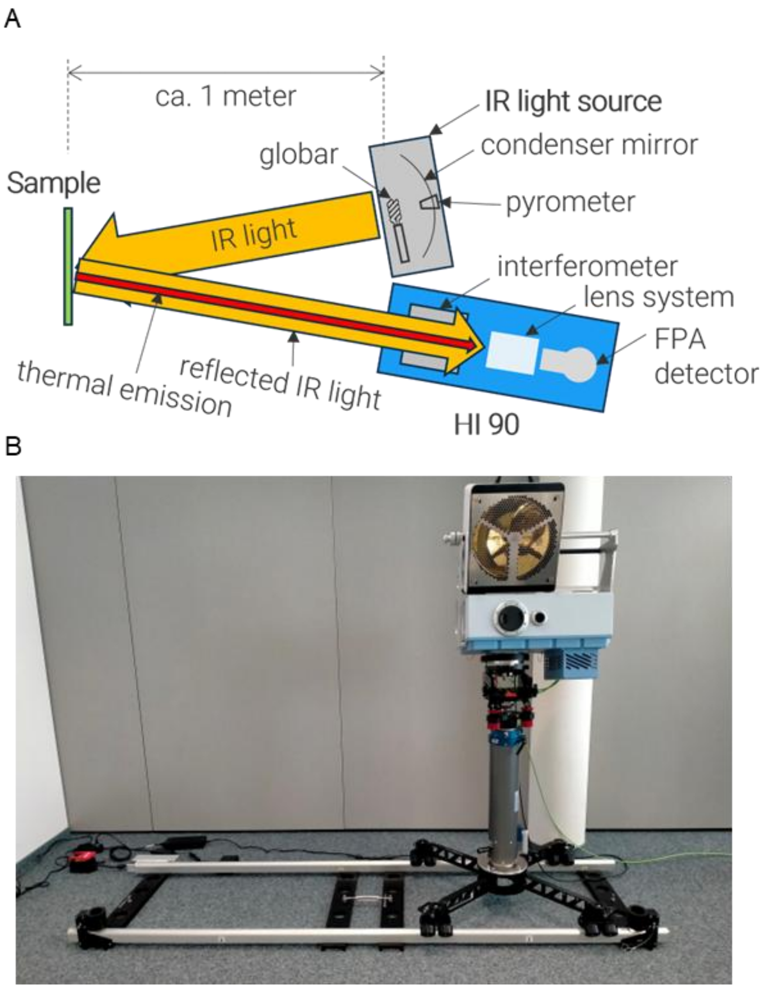

Fig. S1. Mid-IR hyperspectral imaging

(A) Schematic view of the imager set-up; (B) picture of the whole system consisting of the imager, IR-source, raiser system and track.

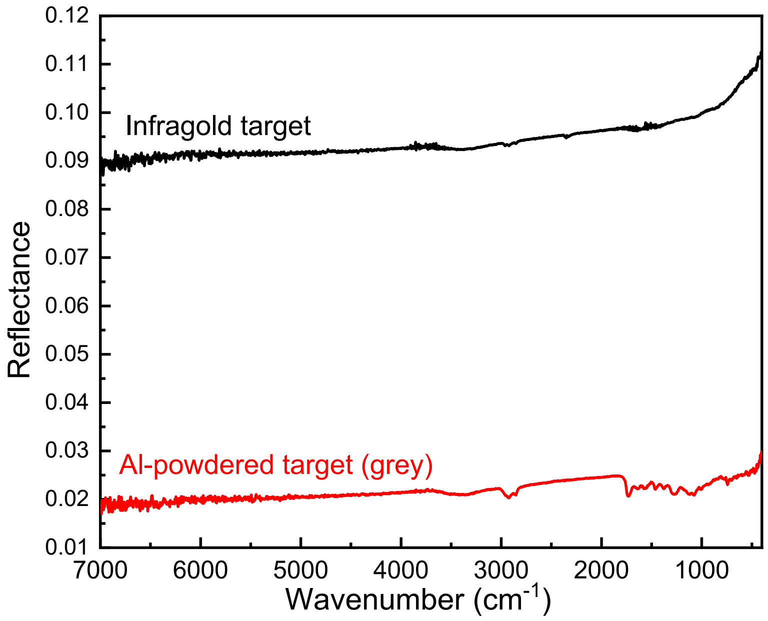

Fig. S2. Reflection to reflectance: reflectance profiles of the reference targets

Reflectance spectra, acquired by the single-point IR spectrometer, in the 7000-800 cm^{-1} range of the two selected reference targets corrected against a flat gold mirror, red line Al-powder target and, black line Infragold target.

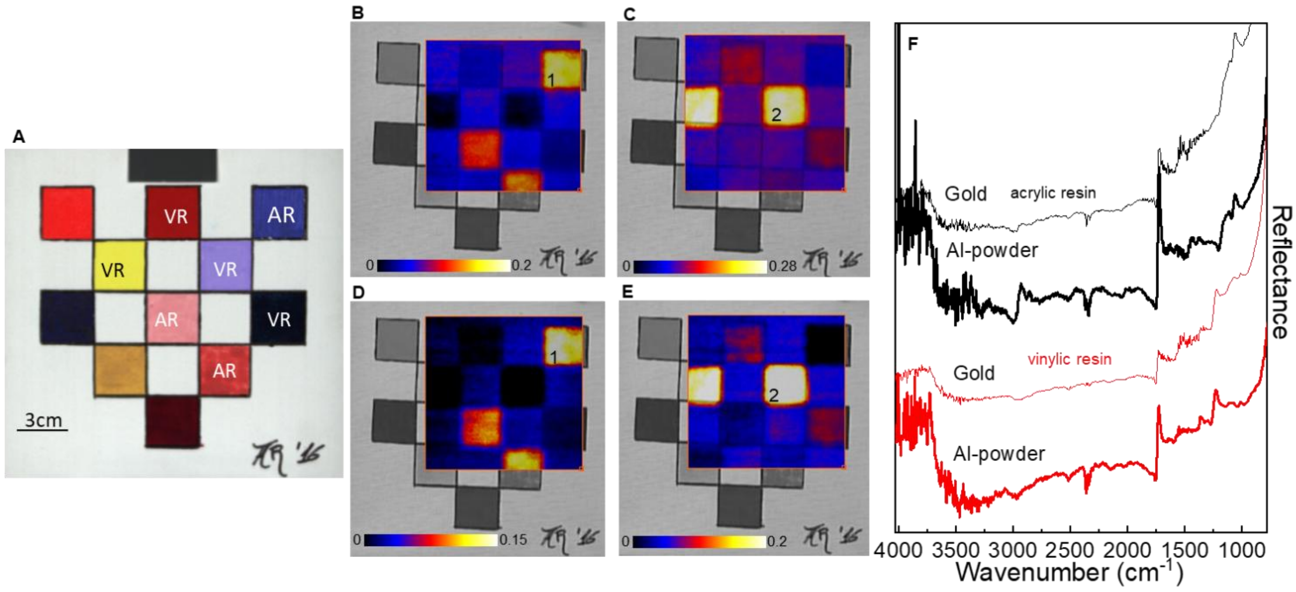

Fig. S3. Reflection to reflectance: comparison of different targets

(A) Picture of the model painting with indication of the binder composition of each sector imaged by the mid-IR camera. AR= acrylic resin, VR= vinyl resin. Maps of the acrylic (difference reflectance 1159 minus 1203 cm^{-1}) and vinylic (difference reflectance 1230 minus 1280 cm^{-1}) binders by the reference target Infragold (B) and (C), and Al-powder (D) and (E); (F) reflectance spectra (3x3 pixel binning) extracted from the maps (sectors 1-acrylic resin, 2-vinylic resin) obtained by using the Infragold (thinner lines, Gold) and Al-powder (thicker lines) targets. Spectra are offset for clarity.

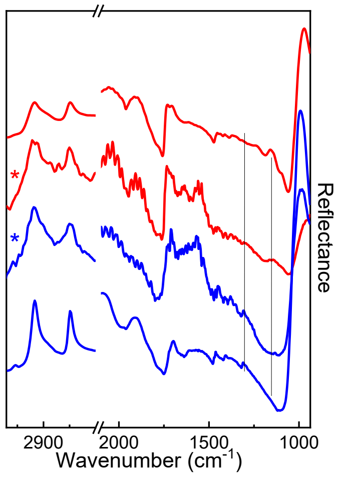

Fig. S4 Mid-IR Hyperspectral Imaging of Model Samples

Reflectance spectra extracted from the hyperspectral data-cube (3x3 pixel binning, * marks) in correspondence of the unaged (red line) and the 100% RH and T=65°C aged (blue line) samples compared to the respective spectra recorded with the portable single point IR spectrometer (blue and red lines without marks). Vertical lines mark the spectral modifications due to the ageing, namely the formation of oxalates (band at 1310 cm^{-1}) and the diminishing of the band assigned to the ν(C-O-C) at ca. 1100 cm^{-1} of the siccative oil.

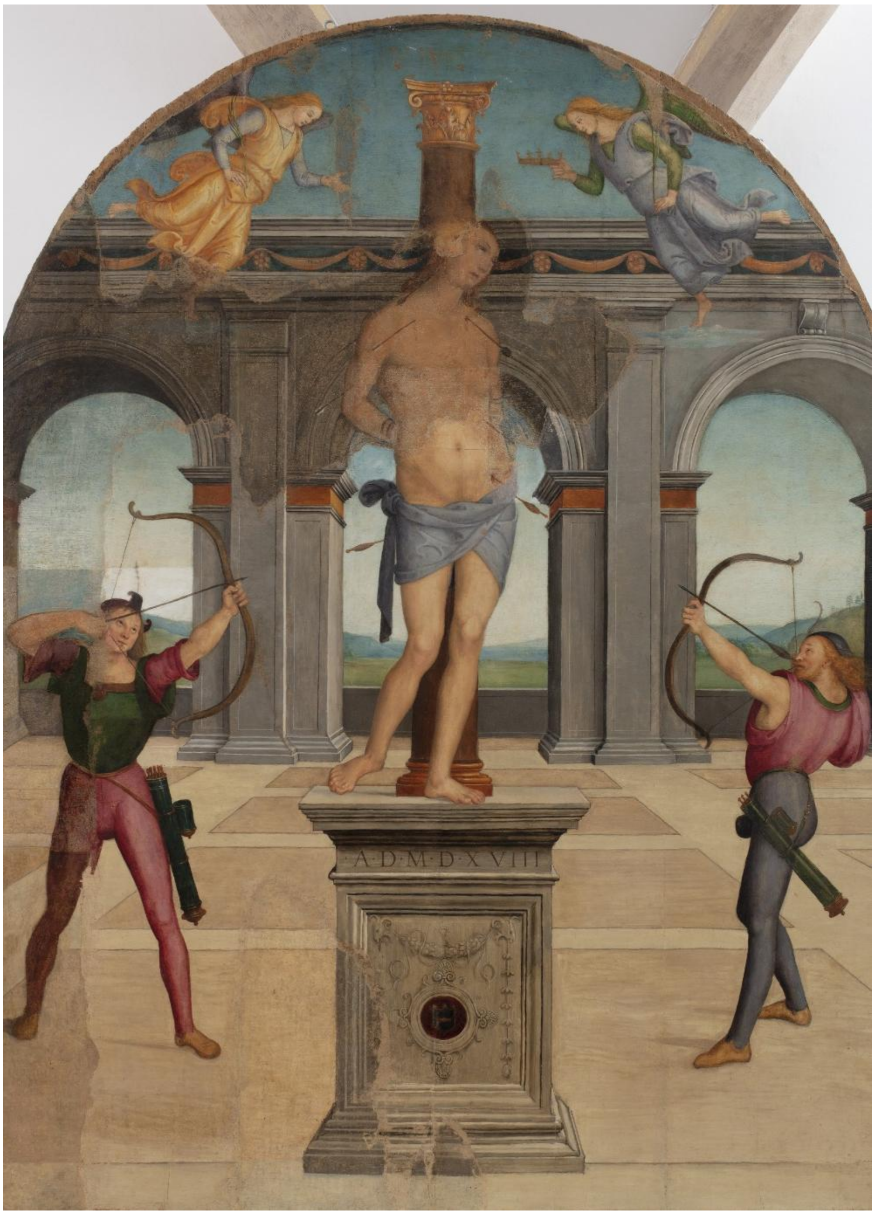

Fig. S5. The Renaissance panel painting

Visible image of a portion of the Renaissance panel painting “*Martirio di San Sebastiano*”, 1518 by Il Perugino conserved at the Galleria Nazionale dell’Umbria.

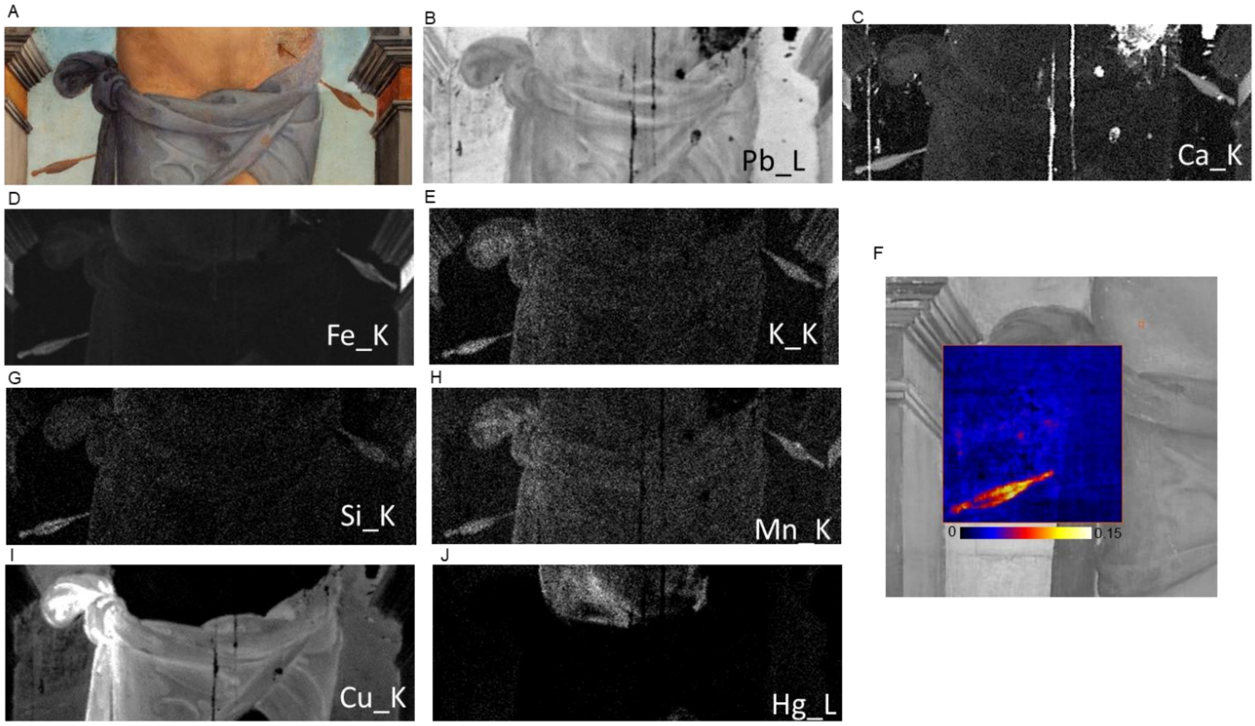

Fig. S6. The Renaissance panel painting: elemental and molecular imaging

(A) Visible image of a detail of the painting; X-ray maps of: (B) lead, Pb(Lα); (C) calcium, Ca(Kα); (D) iron, Fe(Kα); and (E) potassium, K(Kα). (F) mid-IR difference reflectance image in the characteristic spectral ranges for kaolin [996 minus 1046 cm^{-1}, ν_{as}(Si-O)], spectrum shown in fig. S6. X-ray maps of: (G) silicon, Si(Kα); (H) manganese, Mn(Kα); (I) copper, Cu(Kα); and (J) mercury, (Lα).

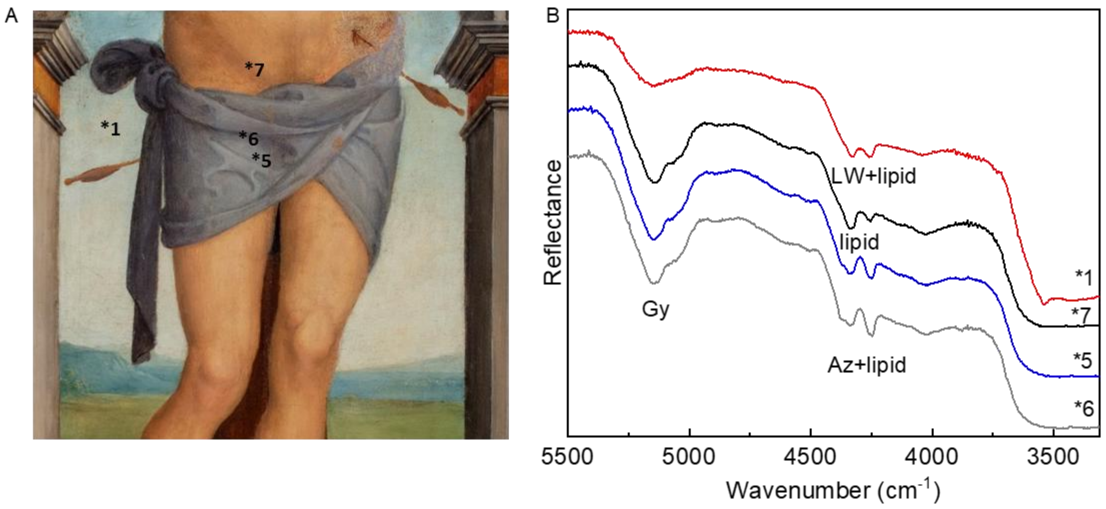

Fig. S7. The Renaissance panel painting: IR reflectance spectra

(A) Visible image of a detail of the painting with indicated the position of the IR single-point measurements shown in (B); (B) External reflection spectra (SWIR range) acquired by the single-point spectrometer in correspondence of the points shown in (A). Spectra are offset for clarity.

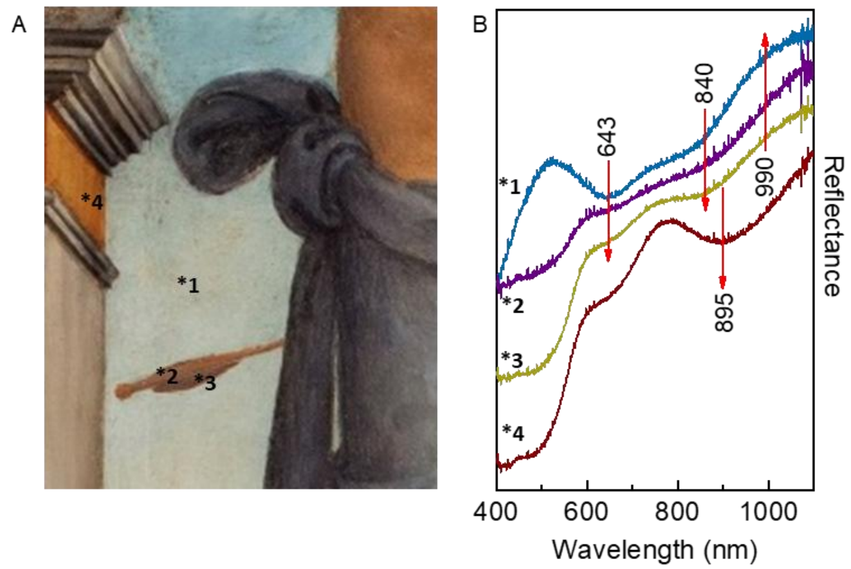

Fig. S8. The Renaissance panel painting: VIS-NIR reflectance spectra

(A) Visible image of a detail of the painting with indicated the position of the VIS-NIR reflectance spectra shown in (B); (B) VIS reflectance spectra (single-pixel extracted from the VIS-NIR hyperspectral cube) in correspondence of the points shown in (A) representative of: *1 azurite in the sky; 2*and 3* hematite (ochre paint) and azurite for the shadow in the arrow detail; and 4* hematite (ochre paint) of the architecture. Spectra are offset for clarity.

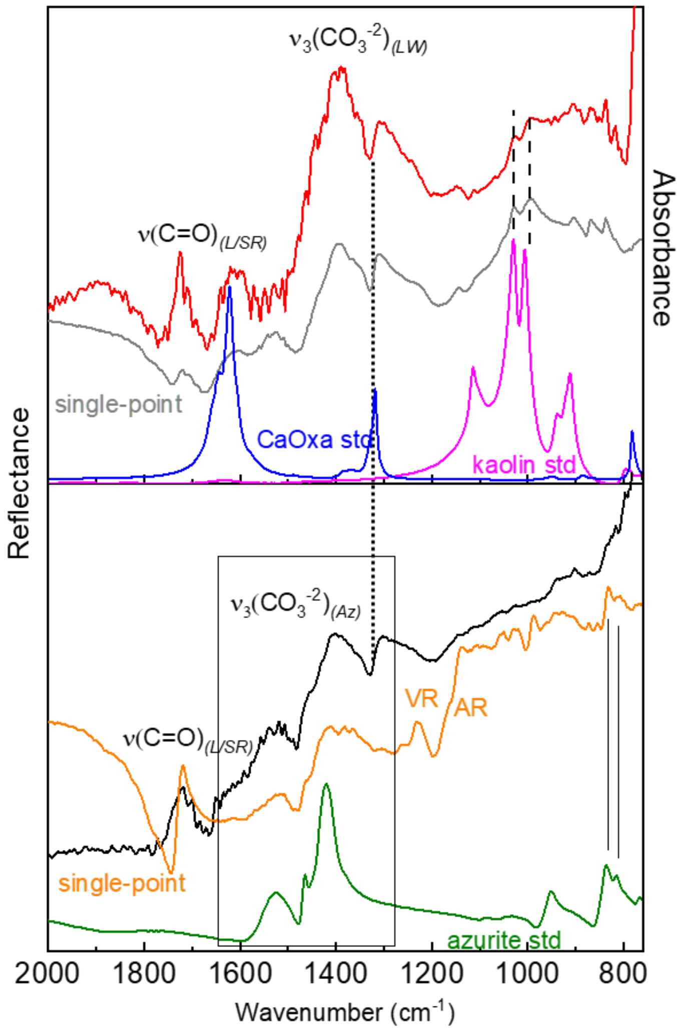

Fig. S9. The Renaissance panel painting: mid-IR reflectance spectra

Reflectance spectra (4x4 pixel binning) extracted from the cube in correspondence of the arrow detail (red line) and the blue-violet loincloth knot (black line). Reflectance spectra acquired by the portable IR spectrometer are also reported (grey line, acquired in the arrow; and orange line, acquired on the blue-violet loincloth, area not completely cleaned). Reference spectral profiles of azurite (green line, reflectance spectrum), kaolin and Ca-oxalate (magenta and blue lines, respectively, absorbance spectrum) are also shown. Spectra are offset for clarity. L= lipids; SR= synthetic resin; AR= acrylic resin, VR= vinyl resin
